# Ghost chest tube after talc pleurodesis

**DOI:** 10.1002/ccr3.3084

**Published:** 2020-07-08

**Authors:** Takeshi Matsumoto, Yusuke Kusakabe, Naoki Yamamoto, Kensaku Aihara

**Affiliations:** ^1^ Department of Respiratory Medicine Saiseikai‐Noe Hospital Osaka Japan

**Keywords:** calcification, chest tube, malignant pleural effusion, talc pleurodesis

## Abstract

After the talc pleurodesis, CT showed the tract made from the chest tube even after its removal. The unexpanded thoracic space might also contribute to it; thus, gathering specific medical history is important to understand this rare phenomenon.

## Graphical abstract

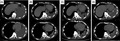

## 1 INTRODUCTION

Talc pleurodesis is widely used to alleviate malignant pleural effusion. We herein present a case of a “ghost” chest tube by talc pleurodesis, which was possibly caused by acute calcific depositions and an unexpanded thoracic space.

**FIGURE 1:**
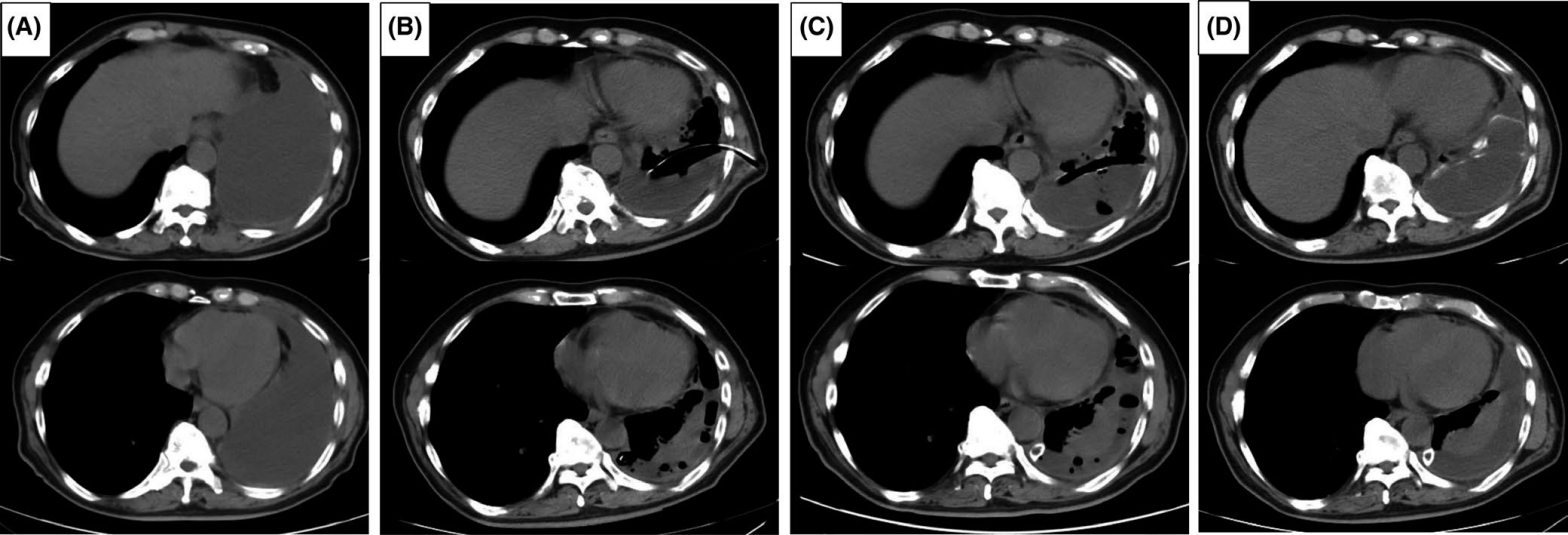
(A) Computed tomography (CT) performed before the drainage showing left pleural effusion. (B) CT after the drainage showing insertion of an actual chest tube. (C) CT performed 1 wk after talc pleurodesis showing the “ghost” chest tube. (D) CT 2 mo after pleurodesis showing the remaining “ghost” chest tube

A 72‐year‐old man was referred to our hospital and diagnosed with advanced lung adenocarcinoma with left malignant pleural effusion Figure 1A. Due to dyspnea upon exertion, the left malignant pleural effusion was drained using a 20‐Fr chest tube Figure 1B. Seven days after the insertion, pleurodesis with 4 g of talc was performed, although the lower lung field could not be fully expanded. After the pleurodesis, computed tomography showed the tract made from the chest tube even after it was removed Figure 1C. This “ghost” chest tube remained for over 2 months after pleurodesis Figure 1D. Talc pleurodesis is widely used to alleviate malignant pleural effusion. However, talc pleurodesis‐induced change frequently remains, which shows high uptake of fluorodeoxyglucose on positron emission tomography,^1^ mimicking malignancy.^2^ Talc is an inflammatory agent that can cause chronic granulomatosis with prolonged immune stimulation due to foreign bodies,^2^ therefore, calcific formation is usually a slow process.^1^ This ghost chest tube, which was possibly caused by acute calcific deposits, is a rare and an interesting clinical presentation, with the possible contribution of the unexpanded thoracic space,therefore, gathering specific medical history is important to understand this rare phenomenon.

## CONFLICTS OF INTEREST

The authors state that they have no conflicts of interest. This research did not receive any specific grant from funding agencies in the public, commercial, or not‐for‐profit sectors.

## AUTHOR CONTRIBUTIONS

TM: prepared the manuscript and reviewed the literature. YK, NY, and KA: critically revised the manuscript and approved the final version of the manuscript.

## ETHICAL STATEMENT

This study was in accordance with the 1964 Helsinki Declaration and its later amendments or comparable ethical standards.

## Data Availability

The data are available upon reasonable request.
